# Supplementary material for: The Mechanism of Houttuynia cordata Embryotoxicity Was Explored in Combination with an Experimental Model and Network Pharmacology
Source: Toxins (Basel). 2023 Jan 13;15(1):73. doi: 10.3390/toxins15010073 (PMC9864403; doi:10.3390/toxins15010073)
Supplement: Supplementary file 1 [file toxins-15-00073-s001.zip › toxins-2145961-supplementary.pdf]

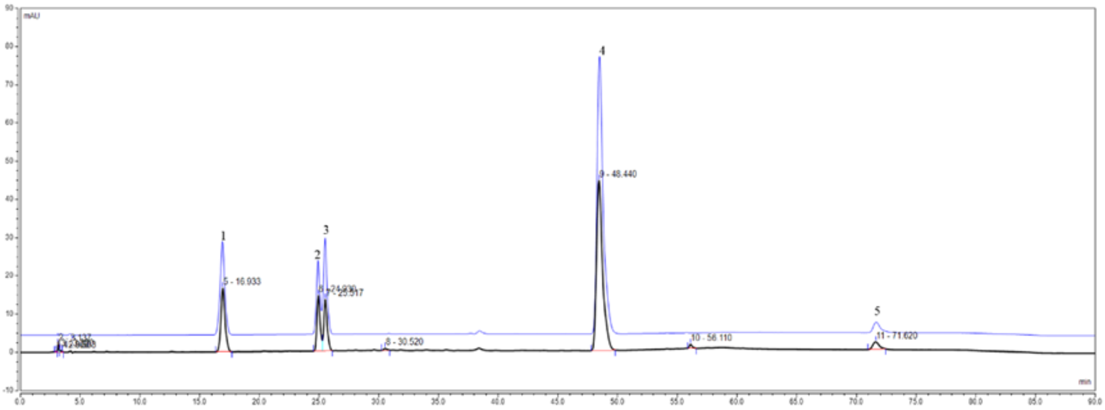

Figure S1: HPLC fingerprint of the aqueous extract of *H.cordata*.Peak 1: neochlorogenic acid; Peak 2: chlorogenic acid; Peak 3: cryptochlorogenic acid Peak4: Hypericin; Peak5: Quercetin Identification of H. cordata extract components by HPLC.
